# Saúde mental na Argentina e no Brasil: avanços e desafios a partir de experiências subnacionais em Río Negro e no Ceará

**DOI:** 10.1590/0102-311XPT072224

**Published:** 2025-05-19

**Authors:** Lucía Belén Perez, Nicolas Gustavo Souza Costa, Francesca Elizabeth Cristina Araújo Bezerra, Amanda Pinheiro, Ricardo José Soares Pontes, Carmem Emmanuely Leitão Araújo

**Affiliations:** 1 Programa de Pós-graduação em Saúde Pública, Universidade Federal do Ceará, Fortaleza, Brasil.; 2 Faculdade de Medicina, Universidade Federal do Ceará, Fortaleza, Brasil.

**Keywords:** Saúde Mental, Política de Saúde, Reforma dos Serviços de Saúde, Mental Health, Health Policy, Health Care Reform, Salud Mental, Política de Salud, Reforma de la Atención de Salud

## Abstract

As políticas de saúde mental se mostram plurais com estratégias, processos e resultados distintos de reformas psiquiátricas nos países da América Latina. O objetivo do artigo foi analisar os avanços e desafios nas políticas de saúde mental na Argentina e no Brasil, considerando experiências subnacionais de reformas. Recorreu-se a um desenho de pesquisa qualitativo, que incluiu um exercício descritivo, interpretativo e comparativo. Além de análise documental, foram realizadas 35 entrevistas com gestores nacionais e da rede de saúde mental de Río Negro, província argentina, e Ceará, estado brasileiro. Os resultados apontam que marcos legais, contextos políticos e características estruturais nacionais circunscrevem as experiências subnacionais. O protagonismo local se manifesta com a formulação e implementação de serviços de atenção à saúde mental com orientação comunitária. O porte das cidades é um fator relevante para a implementação de políticas. Os dois casos têm dificuldades relacionadas à formação dos profissionais, às condições de trabalho, ao financiamento das políticas, bem como à coordenação e integração de serviços da rede de atenção. As políticas estudadas são permeadas por resistências e, por vezes, retrocessos que podem afetar os avanços conquistados nas últimas décadas. Conclui-se que existem similaridades nos casos analisados, diante de condicionantes contextuais e estruturais particulares.

## Abstract (en)

Mental health policies in Latin America are diverse, with different strategies, processes, and outcomes in their psychiatric reforms. This article aimed to analyze the progress and challenges of mental health policies in Argentina and Brazil, considering subnational reform experiences. A qualitative research design was used, including a descriptive, critical, and comparative approach. In addition to documental analysis, 35 interviews were conducted with national policymakers and members of the mental health network in Río Negro Province (Argentina) and Ceará State (Brazil). The results pointed out that legal frameworks, political contexts, and structural characteristics shape the studied cases. Local leadership is evident in the formulation and implementation of community-oriented mental health care services. City size is a relevant factor in policy implementation. Both experiences face challenges related to professional training, working conditions, policy funding, as well as the coordination and integration of health care network services. The studied policies endure resistance and, at times, setbacks that may impact the progress achieved in recent decades. The study concludes that there are similarities between the cases analyzed, influenced by specific contextual and structural factors.

## Resumen

Las políticas de salud mental en América Latina presentan estrategias, procesos y resultados diversos de sus reformas psiquiátricas. El objetivo de este artículo fue analizar los avances y desafíos en las políticas de salud mental en Argentina y Brasil, considerando experiencias subnacionales de reforma. Se utilizó un diseño de investigación cualitativo, que incluyó un ejercicio descriptivo, crítico y comparativo. Además del análisis documental, se realizaron 35 entrevistas con gestores nacionales y con integrantes de la red de salud mental de Río Negro (Argentina) y Ceará (Brasil). Los resultados indican que los marcos legales, los contextos políticos y las características estructurales circunscriben los casos estudiados. Se observa protagonismo local en la formulación e implementación de servicios de atención en salud mental con orientación comunitaria. El tamaño de las ciudades es un factor relevante en la implementación de políticas. Ambas experiencias presentan dificultades relacionadas con la formación de profesionales, las condiciones laborales, la financiación de las políticas, así como la coordinación e integración de los servicios en la red de atención. Las políticas estudiadas enfrentan resistencias y, en ocasiones, retrocesos que pueden afectar los avances logrados en las últimas décadas. El estudio concluye que existen similitudes entre los casos analizados, influidas por factores contextuales y estructurales específicos.

## Introdução

Os países da América Latina revelam elevada carga de sofrimentos mentais, de modo a exigir investimentos em práticas assistenciais no campo da saúde mental ^1^. Embora tenham se desenvolvido a partir de ideias e marcos comuns de reorientação de serviços psiquiátricos, a formulação e a implementação de política de saúde mental nos países da América Latina mostram-se plurais, sendo possível identificar estratégias, processos e resultados distintos ^2^ ^,^ ^3^.

Há estudos que confrontam contextos e estratégias diferentes adotadas em diferentes países da região ^4^ ^,^ ^5^, porém ainda é preciso análises atualizadas capazes não somente de desvendar melhor os caminhos das políticas públicas em saúde mental, mas também gerar informações para novos delineamentos delas. Conjugam-se análises importantes relacionadas às visões dos usuários do sistema, dos profissionais e gestores atuantes nos serviços acerca das políticas e assistência psicossocial ^6^ ^,^ ^7^ ^,^ ^8^ ^,^ ^9^ ^,^ ^10^ ^,^ ^11^. Contudo, ainda é preciso avançar em pesquisas de natureza comparativa sobre a saúde mental nos países da América Latina ^12^.

Desde a década de 1960, Argentina e Brasil se destacam na recepção de ideias que incentivavam a reformulação nos modos de intervir nos casos de transtornos mentais. Os dois países também foram relevantes na formulação e implementação de serviços de bases comunitárias, fruto de coalizões de defesa formadas por movimentos sociais e grupos de interesse, especialmente a partir da década de 1990. Há, entretanto, diferentes trajetórias na implementação de políticas nacionais e dificuldades na institucionalização das políticas em âmbito subnacional ^13^.

Diante da relevância de ampliar esta discussão, este artigo analisa os avanços e desafios nas políticas de saúde mental na Argentina e no Brasil, considerando experiências de Río Negro (Argentina) e do Ceará (Brasil). A análise de experiências em saúde mental nos governos subnacionais favorece compreender não apenas características específicas, padrões institucionais e contextuais, mas também olhares comparados sobre as políticas públicas adotadas.

## Metodologia

Realizou-se uma investigação com desenho qualitativo, mediante o uso de fontes mistas de informações, que inclui exercício descritivo, crítico e comparativo. O método comparativo caracteriza-se por ser compreensivo e diz respeito ao uso de comparações entre um número de casos moderado a fim de inferir sobre alguns fatores comuns a eles. Conjuntamente, foi adotado enfoque analítico hermenêutico-dialético como meio para a compreensão das informações construídas com os sujeitos da pesquisa ^14^.

O intervalo temporal analisado considera o início as primeiras iniciativas de reformas psiquiátricas nos dois países, ainda na década de 1990, enfatizando a última década, na qual se comporta um período de mudanças relevantes na área da saúde mental, tanto na Argentina quanto no Brasil.

Para conhecer alguns pontos de convergência e divergência entre os dois casos, foi realizada revisão de literatura, bem como análise documental, na qual se considerou 32 documentos. Esses foram extraídos das páginas web dos ministérios de saúde, como leis, boletins, comunicações oficiais, tanto a nível nacional, provincial, estadual ou municipal.

O desenho da pesquisa imergiu em experiências e contextos particulares de implementação de políticas de saúde mental na Província de Río Negro e no Estado do Ceará, escolhidos pelo pioneirismo e destaque em matéria de políticas públicas de atenção psicossocial nos seus países, bem como pelo critério de conveniência. A Província de Río Negro, da Patagônia, no sul argentino, é constituída por 77 municípios, com capital em Viedma. O Ceará, estado do Nordeste do Brasil, compreende 184 municípios, sendo Fortaleza a capital.

Os sujeitos da pesquisa foram: (1) gestores com atuação local em serviços da rede de atenção à saúde mental de Río Negro e do Ceará; e (2) atores de referência no âmbito nacional, pelo potencial de contribuir acerca do contexto e desenvolvimento das políticas de cada país (Quadro 1).

**Quadro 1:** Entrevistados por âmbito de atuação, país, período de atuação e vinculação institucional ou serviço de saúde.

ENTREVISTADOS	ÂMBITO DE ATUAÇÃO (PAÍS)	PERÍODO DA ATUAÇÃO NA REDE DE ATENÇÃO À SAÚDE	INSTITUIÇÃO OU SERVIÇO DE SAÚDE
E.1	Nacional (Argentina)	2013-atual	Asociación Argentina de Salud Mental
E.2	Nacional (Argentina)	2005-2009	Atividade mais destacada em relação ao desenvolvimento de políticas de saúde mental na Cámara de Diputados de la Nación
E.3	Nacional (Argentina)	2018-2020	Ministerio de Salud de la Nación
E.4	Nacional (Brasil)	2001-atual	Movimento Nacional da Luta Antimanicomial
E.5	Nacional (Brasil)	1991-2011	Ministério de Saúde
E.6	Estadual (Brasil)	1990-1994	Atividade mais destacada em relação ao desenvolvimento de políticas de saúde mental na Subsecretaria de Direitos Humanos
E.7	Estadual (Brasil)	2005-atual	Fórum Cearense da Luta Antimanicomial
E.8	Estadual (Brasil)	1999-2000	Atividade mais destacada em relação ao desenvolvimento de políticas de saúde mental na Sociedade Brasileira de Psiquiatria, Neurologia e Higiene Mental
E.9	Nacional (Brasil)	1987-atual	Delegação de Supervisão dos Serviços de Saúde
E.10	Nacional (Argentina)	2013-atual	Órgano Nacional de Revisión de Salud Mental
E.11	Nacional (Argentina)	2019-2021	Asociación de Psiquiatras Argentinos
E.12	Estadual (Brasil)	2018-atual	Centro de Atenção Psicossocial − Geral Iguatu
E.13	Estadual (Brasil)	1993-atual	Hospital Psiquiátrico − Fortaleza
E.14	Estadual (Brasil)	2015-2021	Conselho Estadual de Saúde
E.15	Provincial (Argentina)	2020-atual	Hospital Geral − El Bolsón
E.16	Provincial (Argentina)	2020-atual	Centro de Atención Primaria a la Salud − San Carlos de Bariloche
E.17	Provincial (Argentina)	2014-atual	Hospital Geral − Viedma
E.18	Estadual (Brasil)	2019-atual	Centro de Atenção Psicossocial − Geral Fortaleza
E.19	Estadual (Brasil)	1991-atual	Centro de Atenção Psicossocial − AD Quixadá
E.20	Estadual (Brasil)	2016-atual	Centro de Atenção Psicossocial − AD Quixadá
E.21	Estadual (Brasil)	2016-atual	Centro de Atenção Psicossocial − Geral Quixadá
E.22	Estadual (Brasil)	2006-2011	Centro de Atenção Psicossocial − AD Quixadá
E.23	Estadual (Brasil)	2011-2016	Centro de Atenção Psicossocial − AD Quixadá
E.24	Estadual (Brasil)	2020-atual	Centro de Atenção Psicossocial − Infantil Canindé
E.25	Estadual (Brasil)	2018-atual	Centro de Atenção Psicossocial − Geral Canindé
E.26	Provincial (Argentina)	2002-atual	Centro de Atención Primaria a la Salud − Viedma
E.27	Provincial (Argentina)	1998-atual	Centro de Atención Primaria a la Salud − San Carlos de Bariloche
E.28	Estadual (Brasil)	2002-atual	Centro de Atenção Psicossocial − AD Canindé
E.29	Estadual (Brasil)	2019-atual	Centro de Atenção Psicossocial − AD Fortaleza
E.30	Provincial (Argentina)	1991-atual	Hospital Geral − El Bolsón
E.31	Provincial (Argentina)	2018-atual	Hospital Geral − San Carlos de Bariloche
E.32	Estadual (Brasil)	2021-atual	Centro de Atenção Psicossocial − Infantil Iguatu
E.33	Estadual (Brasil)	2018-atual	Centro de Atenção Psicossocial − Geral Iguatu
E.34	Provincial (Argentina)	2014-2022	Centro de Atención Primaria a la Salud − El Bolsón
E.35	Provincial (Argentina)	2007-atual	Centro de Atención Primaria a la Salud − San Carlos de Bariloche

Fonte: elaboração própria.

Ao diferenciar volume e riqueza de informações, o trabalho utilizou a concepção de *information power* ^15^. A técnica selecionada para a conformação da amostra foi “bola de neve ou cadeia” ^16^. Os primeiros participantes foram selecionados a partir da sua relevância no desenvolvimento das políticas públicas de saúde mental. Pedia-se a eles indicações de outros atores relevantes para atender os objetivos da pesquisa; do mesmo jeito, foram incluídos atores relevantes nas cidades selecionadas, especialmente a partir de visitas nos serviços de atenção especializada.

Foram realizadas 35 entrevistas entre agosto de 2021 e novembro de 2022, com total de 35 entrevistas. Os gestores de serviços tinham atuação local nas cidades rionegrinas: Viedma, San Carlos de Bariloche e El Bolsón; e nas cidades de Fortaleza, Quixadá, Canindé e Iguatu, no Ceará. A escolha das cidades considerou a relevância dos dispositivos das cidades na história da construção da saúde mental em cada um dos países e a cobertura da distribuição espacial na região. Todas as cidades selecionadas foram visitadas pela pesquisadora principal.

Os roteiros das entrevistas, incluíram dois eixos: caracterização da rede de saúde mental e mudanças nas políticas públicas, considerando avanços e desafios. Entrevistas de sujeitos argentinos foram realizadas em espanhol, com posterior tradução para português pela equipe da pesquisa. Doze entrevistas foram realizadas de forma virtual, pela plataforma Google Meet (https://meet.google.com/landing?pli=1).

A análise das informações foi realizada com o software Atlas T.I (http://atlasti.com/), estabelecendo categorias e códigos de análise.

Seguiu-se a *Resolução do Conselho Nacional de Saúde* (CNS) *nº 466/2012*, com disposição de Termo de Consentimento Livre e Esclarecido (TCLE) aos participantes, com apreciação do Comitê de Ética da Pró-Reitoria de Pesquisa e Pós-graduação da Universidade Federal do Ceará (parecer nº 5.124.298).

## Resultados

### Marcos legais, contexto e características estruturais

Características estruturais dos sistemas de saúde condicionam mundanças em políticas públicas específicas. Municípios argentinos possuem funções administrativas marginais diante das atribuições provinciais, ao passo que, no Brasil, o percurso de descentralização agregou maior autonomia aos municípios ^17^. Apesar disso, o Brasil detém mecanismos de coordenação federativa com maior capacidade de implementação de políticas nacionais de base local ^18^.

A Argentina apresenta um subsistema público em saúde marcado por um corporativismo segmentado ^17^, visto a fragmentação “*organizacional, territorial, financeira e, consequentemente, de direitos*” ^19^ (p. 2) associada ao fortalecimento do mercado no setor saúde. Já no Brasil, instituiu-se um sistema público universal, concomitante a um sistema privado fortalecido, de modo a persistir uma cobertura assistencial duplicada para um quarto da população beneficiária de planos e seguros privados de saúde ^20^. 

Em ambos os casos, experiências subnacionais protagonizaram as reformas psiquiátricas, como em Río Negro e no Ceará. 

**Figura 1:**
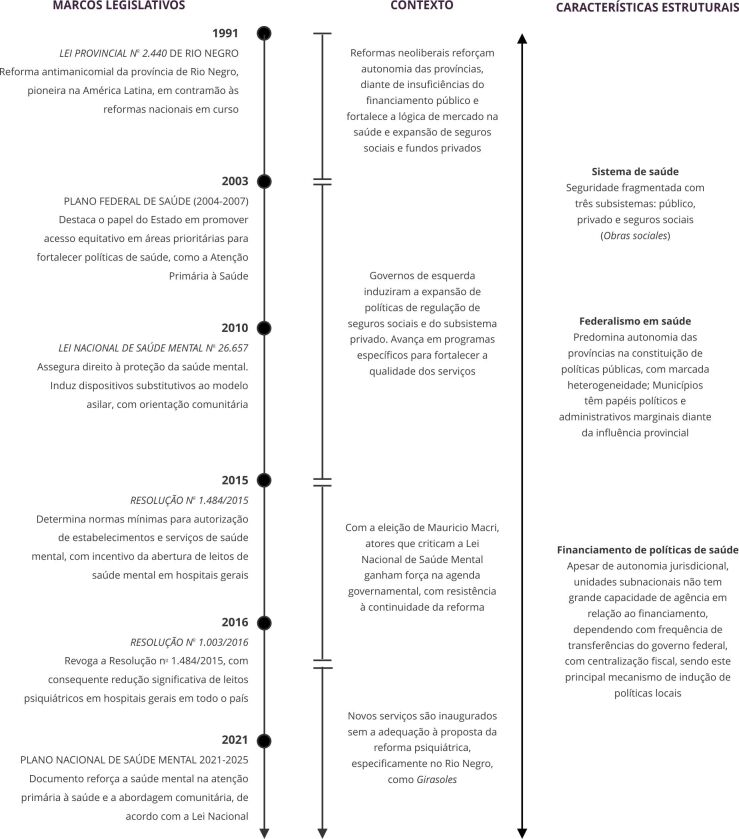
Marcos legais, contexto e características estruturais da Política de Saúde Mental que circunscrevem a experiência na província de Río Negro, Argentina, entre 1991 e 2021.

As Figuras 1 e 2 fazem uma síntese dos principais marcos legais, dos contextos e da estrutura que circunscrevem as experiências de Río Negro, e do Ceará, respectivamente.

Fonte: elaboração própria.

**Figura 2:**
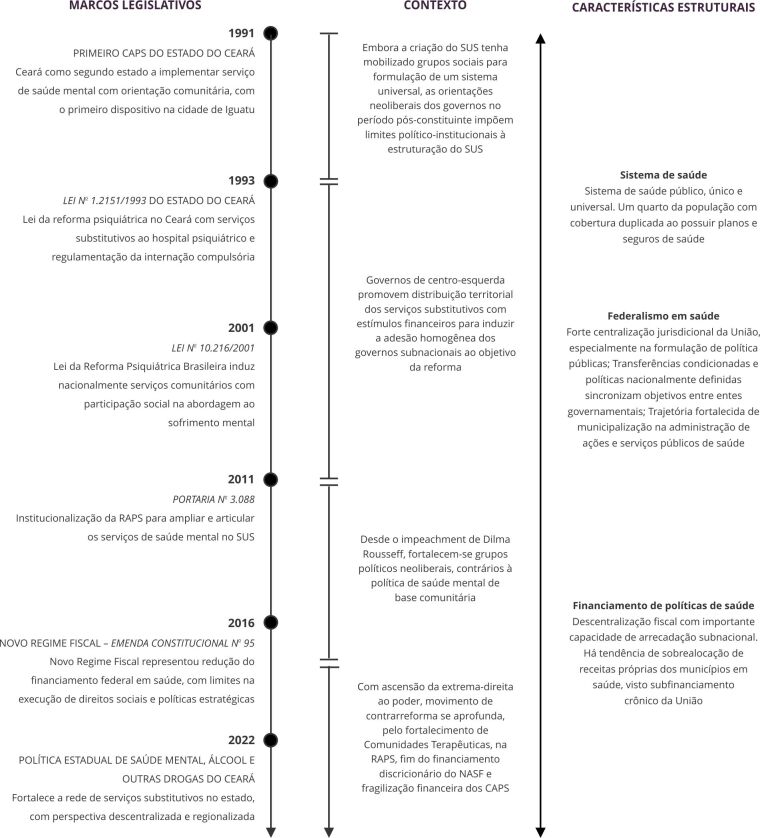
Marcos legais, contexto e características estruturais da Política de Saúde Mental que circunscrevem a experiência no Estado do Ceará, Brasil, entre 1991 e 2022.

CAPS: Centros de Atenção Psicossocial; NASF: Núcleo Ampliado de Saúde da Família; RAPS: Redes de Atenção Psicossocial; SUS: Sistema Único de Saúde. Fonte: elaboração própria.

Importante destacar que a Lei da Reforma Psiquiátrica Brasileira, instituída em 2001, antecede quase uma década a Lei Nacional de Saúde Mental Argentina, sancionada apenas em 2010 ^21^. Nos dois casos, registra-se a indução política e financeira ao longo do tempo, associados aos contextos governamentais que favorecem ou restringem processos de mudanças nas políticas nacionais. As leis subnacionais de Río Negro e do Ceará já apontavam para reformas na Argentina e no Brasil antes de marcos normativos nacionais. Nos dois casos subnacionais se previa a extinção progressiva de hospitais psiquiátricos especializados, públicos ou privados, com a orientação de constituição de rede de serviços substitutiva.

### A implementação de políticas de saúde mental

A história de Río Negro se conformou como uma exceção no país, tendo, “*em 1990 a sanção de uma lei, que é a 2.440 do Río Negro, de desmanicomialização, que ficou como reduto místico e romântico do que foi a reforma, que teve grande repercussão no sul do nosso país, mas não se estende por diferentes motivos*” (E.10).

As mudanças sociais e culturais relacionadas à implementação e consolidação de políticas dificultam retrocessos ao longo do tempo. Considera-se, por exemplo, “*que o Río Negro tem hoje* (...)*: ver como desumano as pessoas voltarem para um hospital psiquiátrico*” (E.15).

Vinte anos após a lei provincial de Río Negro, foi estabelecida a legislação argentina, como fruto da coalizão de movimentos como “La Colifata”, “Frente de Artistas del Borda” e outros grupos “*que acabaram se unindo num único coletivo em torno da lei, porque, além disso, a lei foi discutida por esses grupos e houve contribuições*” (E.2). A política nacional passou a respaldar a implementação de novas práticas, orientadas a um modelo psicossocial, substitutivo à lógica manicomial, embora com fragilidades.

O porte das cidades apareceu como um fator relevante para a implementação de políticas. Segundo os participantes, as cidades menores permitem uma abordagem mais comunitária, interdisciplinar e singular das pessoas que precisam de cuidado, conforme visto em El Bólson. As características dessa cidade pequena permitiram o desenvolvimento de práticas pioneiras em saúde mental.

Nos últimos anos, a sustentação de novas práticas parece ter se tornado mais difícil. No caso de San Carlos de Bariloche, por exemplo, muitas pessoas relataram que a cidade aparenta “*continuar tendo um modelo psiquiátrico*” (E.15). Um fato destacado diz respeito à abertura, durante o período de pandemia de COVID-19, de um centro de internações por motivo de saúde mental, não inserido no hospital geral. Embora o motivo da decisão se justifique pela crise sanitária, preocupações foram levantadas em função da permanência desse equipamento sem uma regulamentação e clareza de sua função na rede de atenção à saúde.

No caso brasileiro, ressaltam-se importantes resistências de atores e coletivos que atuam em âmbito nacional pela Reforma Psiquiátrica Brasileira: “*A reforma psiquiátrica está se mantendo e está resistindo à tentativa de aniquilamento e de retorno ao modelo passado, porque há uma significativa, uma bastante satisfatória presença dos movimentos sociais*” (E.6).

Particularmente no Ceará, à semelhança do observado na província argentina, a implementação de políticas também foi percebida com maior força nos municípios de pequeno e médio porte. A capital Fortaleza, que concentra quase dois milhões e meio de habitantes, mostrou-se menos permeável à implementação de alternativas aos hospitais psiquiátricos: “*Fortaleza, com vários hospitais psiquiátricos tradicionais, oferecia uma certa resistência para a implantação dos CAPS*” (E.19).

Os Centros de Atenção Psicossocial (CAPS) assumiram caráter estratégico na reorientação do modelo de atenção à saúde mental no estado, mediante uma expressiva expansão na década de 1990 e 2000, especialmente nas cidades menores.

### Formação dos profissionais e condições de trabalho: uma lacuna

Os paradigmas que orientam a formação de atores na área de saúde mental nos dois países não acompanharam os avanços quanto aos conteúdos normativos das políticas públicas, tornando-se um ponto crítico de resistência às mudanças. A formação acadêmica, em termos de desenvolvimento de competências para atuação em modelos de base territorial, não asilar, nos dois países, tornou-se um ponto crítico, incidindo em resistência às reformas.

No caso argentino, práticas contraditórias aos objetivos reformistas são observadas. Para alguns, processos formativos pautados pela lógica manicomial persistem: “*não podemos esperar que mais psiquiatras comunitários apareçam se suas residências são feitas em um hospital psiquiátrico*” (E.3). Esse modelo de formação se reflete nas maneiras de lidar com as necessidades de saúde da população. Um exemplo disso se dá na definição da agenda das equipes de saúde, ainda priorizando abordagens individuais: “*falta formação para compreender que a abordagem dos sofrimentos mentais vai muito além do dispositivo da psicoterapia, mas sim trabalhar em rede*” (E.30).

Problemas de formação se somam à precariedade das condições de trabalho “*Com hospitais abandonados, com salários baixíssimos, com plantões muito longos e exaustivos, com pouco reconhecimento social*” (E.11).

No Brasil, a qualificação profissional também se mostra um desafio, “*cada vez são mais ligadas ao modelo hegemônico medicalizante*” (E.5). Assim, a baixa compreensão e capacidade em atuar na perspectiva intersetorial e interprofissional favorece dissonâncias entre o idealizado e o efetivado nas práticas de atenção à saúde.

Somado a isso, a alternância governamental contribui com a descontinuidade de práticas inovadoras. As dificuldades se aprofundam com a precarização do trabalho, dado que “*os vínculos estatutários de concursos são a minoria e isso dificulta uma série de processos também, que se traduz na qualidade do serviço* (...), *com o resquício manicomial que claramente existe*” (E.13).

### Financiamento da política de saúde mental: insuficiências

Na Argentina, percebe-se insuficientes mecanismos de vinculação do orçamento público aos objetivos postos à ocasião da formulação da Lei Nacional de Saúde Mental e ao sistema público de saúde em sua totalidade.“*O instrumento que o Ministério da Saúde argentino tem para modificar as práticas nas províncias é orçamentário*” (E.2), mas há indícios de que a falta de recursos econômicos asfixia a proposta de reforma, inclusive impondo risco à sobrevivência dos serviços substitutivos já implementados.

Acrescenta-se, contudo, que o governo federal tem sustentado um modelo menos eficiente e mais oneroso, senão também contrário às premissas da reforma: “*temos hospitais psiquiátricos fortalecidos a partir do orçamento, do número de profissionais, é neles que estão os psicofármacos, ainda são neles as residências*” (E.3). Ou seja, ainda há fomento da manutenção de hospitais monovalentes, de práticas de medicalização, com internações prolongadas, em detrimento de estratégias comunitárias.

Ressaltam-se as particularidades do sistema federativo argentino que outorga autonomia às províncias na implementação de políticas, gerando um clima altamente heterogêneo ao longo do país. Os gestores de Rio Negro identificam a heterogeneidade dos contextos de implementações: “*Não são muitas as províncias que fizeram um forte investimento para mudar o sistema de atendimento agora, mesmo que você tenha o orçamento, você tem outros obstáculos*” (E.2). Em resumo, os mecanismos de indução para políticas nacionais são limitados, com dependência importante de transferências intergovernamentais pelo governo central, sem previsibilidade na decisão de cada governo subnacional aderir ou não às estratégias desenhadas: “*É uma decisão política.* (...) *Tem muitas províncias que já fizeram a adaptação* (...). *Muitos criticaram a lei e pediram para não cumprir a lei, como se ela não pudesse ser cumprida*” (E.1).

Em adição, pensa-se na necessidade de um diagnóstico situacional da saúde mental no país, necessário para a alocação de recursos baseados nas necessidades.

Já no Brasil, especialmente após a ascensão de governos de neoliberais e conservadores, aprofundou-se nas consequências da falta de acréscimo nas receitas orçadas e políticas de austeridade, sufocando progressivamente a qualidade e a capacidade de resposta dos serviços de saúde mental. Assim, apesar de reduzido o financiamento aos leitos em hospitais psiquiátricos, “*há uma escassez de dispositivos desse tipo, que atende emergência, há escassez também de leitos em hospitais clínicos, para dar guarida às comorbidades, então há uma dificuldade estrutural crônica por conta do baixo investimento a longo do tempo...*” (E.13).

Considerando as diferentes responsabilidades das unidades da federação e pactuações intergovernamentais, parece ser relevante fortalecer a coordenação da política municipal: “*não existe remuneração por esse cargo, não existe carga horária reservada para esse cargo*” (E.28).

Quanto ao papel da União, foram citadas as implicações dos diferentes modelos de financiamento implementados no histórico da política. O aprofundamento da crise orçamentária e movimentos de contra-reformas e põem ao encontro das repercussões do Novo Regime Fiscal aprovado em 2016, revogado em 2023. Tal medida é nomeado por um dos sujeitos da pesquisa como “PEC da morte”, visto o congelamento de recursos da saúde e da educação por 20 anos.

Restringe-se, assim, a capacidade de articular respostas em saúde às alterações nas dinâmicas dos territórios.

### Redes de saúde mental: desafios de coordenação e integração

O sistema público de saúde mental da Província de Río Negro organiza-se em seis zonas sanitárias e 36 programas de áreas de hospitais, cada área com um hospital geral de referência, além de 184 Centros de Atenção Primária à Saúde e 22 estruturas intermediárias, incluindo Casas de Meio-Caminho, Centros de Dia, Casas de Convivência, *Centros de Integración Socio Comunitaria de Salud Mental Comunitaria y Adicciones*, entre outros. Além da inclusão de leitos de saúde mental nos hospitais gerais de toda a província, a rede dispõe de Casas de Meio-Caminho, espaços de acolhimento nos quais, após um período de internação, é ofertado apoio para a reinserção comunitária e laboral. Criaram-se, também, centros culturais abertos à comunidade, mas especialmente orientados à atenção à saúde mental. Não há hospitais psiquiátricos monovalentes na rede de saúde mental de Rio Negro. Outro elemento fundamental que possibilitou avanços foi a inclusão na cena da figura de operador comunitário em saúde mental, membros responsáveis pela integração dos serviços com a comunidade, agindo “*como um recurso não convencional daqui do Rio Negro que começou a ser implantado a partir da lei* [provincial], *da necessidade de trabalho comunitário*” (E.15). Embora relevantes para a implementação da política, a quantidade de operadores é percebida como insuficiente diante das necessidades.

Apesar dos avanços na inclusão de serviços de saúde mental em hospitais gerais e Centros de Atenção Primária à Saúde, a implementação da política argentina é marcada pela heterogeneidade entre as províncias. Existe morosidade na efetivação dos serviços, assim como o desequilíbrio entre fechamento de serviços de orientação asilar e a criação de opções de foco comunitário que consigam absorver a demanda derivada desses ajustes nas demais províncias do país.

A criação de serviços na Argentina parece ainda não corresponder às expectativas descritas nas normativas nacionais e provinciais. Além de escassos, eles carecem de coordenação em rede. As respostas assistenciais às necessidades de atenção em saúde mental ainda têm o hospital psiquiátrico como espaço em destaque. Nesse sentido, “*existe uma enorme lacuna entre os problemas prevalentes de saúde mental e a capacidade de resposta do sistema de saúde, porque a concentração da resposta está nos hospitais psiquiátricos e no primeiro nível de atenção*” (E.3).

Ademais, vale sinalizar a discricionariedade das gestões dos serviços agindo em resistência à orientação de desinstitucionalização. Na cidade de San Carlos de Bariloche, por exemplo, é citada nas entrevistas a decisão do hospital geral de manter o funcionamento do serviço *Girasoles*, local de atendimento em saúde mental fora do espaço hospitalar, criado à ocasião da resposta à pandemia de COVID-19. Apesar dos diretores da área de saúde mental do hospital pontuarem que há um processo de adaptação para o tornar uma Casa de Meio-Caminho, para a maior parte dos entrevistados da província não está claro o modo pelo qual se dá as internações e o fluxo entre *Girasoles*, o hospital geral e os demais dispositivos da rede. Essa situação é interpretada como persistência do modelo psiquiátrico.

O desenvolvimento dos pontos de atenção não foi devidamente acompanhado de orientações claras a respeito dos fluxos de funcionamento e de articulação entre componentes da rede ou entre os demais dispositivos do sistema. Persistem desarticulações intersetoriais, visto os atritos com o sistema judicial, as incongruências nos processos formativos e a insuficiência das políticas de inserção no âmbito laboral.

Diversos fatores são entendidos como integrantes da complexidade do problema de desarticulação da rede de saúde mental argentina, como a falta de habilidade de diagnosticar problemas prevalentes, a prática rotineira de encaminhamento para serviços hospitalares psiquiátricos pela equipe da atenção primária, o número insuficiente de profissionais nos serviços de referência, as iniquidades de acesso a psicofármacos, a resistência dos serviços de hospitais gerais em admitirem pacientes com sofrimento mental com critérios de internação, entre outros.

Há ainda reduzida adesão do subsistema privado e das organizações de obras sociais (planos sociais corporativistas) que, por lei, têm a obrigação de atender as demandas psicossociais: “*isso não se cumpre aqui.* (...) *eles já deveriam, há muitos anos, cumprir com essa regulamentação e não cumprem*” (E.31). Isso acarreta problemas de acesso a direitos básicos e superlotação dos serviços públicos.

No Ceará, apenas cerca de 15% da população possui planos privados de assistência médica-hospitalar, o que indica a necessidade de uma rede pública robusta. A rede é disposta por 171 CAPS, seis Consultórios de Rua, 2.927 equipes da Estratégia Saúde da Família, cinco Unidades de Acolhimento, dez Serviços de Residência Terapêutica, 235 leitos em hospitais gerais, três hospitais psiquiátricos, sendo dois filantrópicos conveniados ao Sistema Único de Saúde (SUS). Há avanços recentes no sentido de fortalecer a rede de serviços substitutivos, em perspectiva decentralizada, com mecanismos de articulação regional. A segmentação e desarticulação dos serviços nas unidades municipais se aprofundam diante dos conflitos quanto ao não seguimento de paciente com sofrimento mental leve ou moderado na atenção primária à saúde (APS), sobrecarregando as equipes do CAPS.

A agenda dos profissionais da APS privilegia atendimentos individuais, em detrimento de práticas de matriciamento, reuniões de equipe e intercâmbios interdisciplinares: “*as outras coordenações já não querem liberar muito as equipes do Programa Saúde da Família para vir para as reuniões, você já acaba tendo a dificuldade para ter o dia do matriciamento na agenda deles, porque a gente está muito sobrecarregado e aí isso vai sufocando o processo*” (E.28).

Além disso, mudanças incrementais em políticas estratégicas são entendidas como contrárias às reformas que delinearam a rede de serviços comunitários. Iniciativas de financeirização da saúde fortalecem planos e instituições privadas, ainda resistentes às proposições reformistas. Os mecanismos de abordagem de grupo, de apoio matricial e interdisciplinar com inserção no território estariam fragilizados, inclusive no contexto da pandemia de COVID-19: “*quando a gente mais precisou de educação e saúde, as equipes foram desmontadas*” (E.28).

A Política Nacional sobre Drogas, estabelecida em 2019, é entendida como apoio e incentivo a práticas contrárias à Reforma, na medida em que “*estabelece as práticas manicomiais, violam os direitos das pessoas em sofrimento mental e põe fim à rede de atenção psicossocial, aniquilando a lógica do cuidado em liberdade*” (E.14). Há ainda incorporação de comunidades terapêuticas à rede com fomento às políticas de abstinência, em detrimento de uma abordagem que prioriza a redução de danos.

O artigo argumenta para semelhanças e diferenças entre os casos estudados, identificando muitos desafios comuns, apesar de trajetórias e condições estruturais distintas. O Quadro 2 sistematiza avanços e desafios das experiências em saúde mental no Río Negro e Ceará.

**Quadro 2:** Avanços e desafios das experiências em saúde mental em Río Negro (Argentina), e Ceará (Brasil).

	RÍO NEGRO	CEARÁ
Avanços	Não há hospitais psiquiátricos especializados na rede de saúde mental de Río Negro; Todos os leitos para internações psiquiátricas estão dispostos em hospitais polivalentes; Quase a totalidade dos Centros de Atenção Primária à Saúde dispõe de Operador de Saúde Mental, incorporando o conhecimento comunitário, como interface entre os usuários e o serviço de saúde; Experiência de Río Negro é exceção em relação à heterogeneidade de implementação da política de saúde mental na Argentina, que tem dificuldade de induzir a política mesmo após uma lei nacional.	Implementação de uma rede de atenção à saúde substitutiva relevante e diversificada; Cidades do interior do Estado do Ceará não têm hospitais psiquiátricos especializados, tendo extinção progressiva de hospitais psiquiátricos monovalentes no estado; Há leitos de internação psiquiátrica em hospitais gerais em todas as regiões de saúde; Experiência do Ceará reflete uma trajetória de maior homogeneidade de implementação da política de saúde mental no Brasil, com indução política e financeira do Governo Federal.
Desafios	Subsistema privado e das *Obras sociales* persistem com baixa adesão às orientações da reforma, diante dos limites na regulação desses serviços; Heterogeneidade de implementação da Reforma Psiquiátrica entre as províncias; Processos formativos e práticas de trabalho ainda cristalizados pelo modelo psiquiátrico hegemônico Fluxos assistenciais pouco claros, com lacunas na coordenação e articulação entre serviços; Novos serviços sem adequação às proposições da reforma psiquiátrica foram inaugurados durante a pandemia de COVID-19, com destaque para *Girasoles* em Río Negro; Financiamento insuficiente impõe limites aos serviços substitutivos, com forte dependência das transferências de recursos pelo governo central, que não apresenta condições orçamentárias adequadas para apoiar e ou ampliar a rede psicossocial nas províncias.	SUS implementado, mas com manutenção de planos e instituições privadas, ainda resistentes às orientações reformistas; Fragilidades na RAPS que imprimem dificuldades em garantir atenção com qualidade às demandas de transtorno mental e sofrimento psíquico; Hospitais psiquiátricos especializados continuam compondo a rede complementar à RAPS do Estado do Ceará, com um equipamento público e dois filantrópicos, além de outras alternativas de atenção excludentes incentivadas nas últimas décadas; Dificuldade de a capital Fortaleza instituir uma rede ampla e efetiva de cuidado em saúde mental; Processos formativos e práticas de trabalho ainda cristalizados pelo modelo psiquiátrico hegemônico; Comunidades terapêuticas fortalecidas como ponto de atenção complementar à RAPS, com persistências de práticas manicomiais; Políticas de austeridade recentes com aprofundamento do subfinanciamento crônico pela União restringiu a capacidade de resposta dos governos locais, sobrecarregando receitas próprias.

RAPS: Redes de Atenção Psicossocial; SUS: Sistema Único de Saúde.

Fonte: elaboração própria.

## Discussão

Os contextos sociopolíticos, econômicos e institucionais, as características das políticas de saúde, as configurações federativas, as ações de grupos de interesse e dos governos incidem nas definições e configurações das políticas de saúde mental ^13^.

Como os resultados apontam, as alternativas para a substituição do tratamento manicomial se manifestam mediante dificuldades estruturais, escassez de recursos financeiros e humanos, vinculados a obstáculos políticos na implementação de políticas de saúde mental ^8^ ^,^ ^11^ ^,^ ^22^ ^,^ ^23^.

Na medida em que a Argentina persiste com lentidão na implementação das novas políticas de saúde mental, mesmo após a lei nacional de 2010 ^13^ ^,^ ^24^, o Brasil experimenta reformulações das políticas que se traduzem como retrocessos em sua trajetória de mudanças ^23^ ^,^ ^25^ ^,^ ^26^ ^,^ ^27^.

Em relação à capacidade de implementação das políticas, sabe-se que na Argentina há maior espaço para legislações provinciais e negociação dos padrões de composição de receitas, enquanto no Brasil prevalece a capacidade da União de induzir políticas pactuadas com os demais entes federativos. Dessa forma, no caso argentino, o federalismo favorece maior autonomia para as províncias, por conseguinte, heterogeneidade de parâmetros de implementação de uma rede alternativa aos hospitais monovalentes, gerando vivências diversas ao longo do país e favorecendo desigualdades nas políticas estabelecidas nas regiões do país ^13^ ^,^ ^24^. A experiência na Patagônia, com o caso de Río Negro é pioneira no país, mas com baixa repercussão no resto da Argentina e com grandes disparidades na implementação das diretrizes centrais, conformando uma exceção mais que uma regra ^23^. Já no Brasil, a capacidade da União coordenar políticas públicas e, por conseguinte, incidir nos processos decisórios e nas condições de implementação locais, em consonância com os propósitos de universalização do SUS emprega avanços relativamente homogêneos na rede de atenção psicossocial ao longo do país, apesar da persistência de desigualdades ^25^.

As cidades com menor porte populacional funcionaram como pioneiras, inclinadas a reformas, nas duas unidades subnacionais estudadas. Ao contrário do contexto argentino, que resguarda papéis políticos e administrativos marginais aos municípios diante da influência provincial, no Brasil, a trajetória de descentralização fortalece a unidade municipal na administração de ações e serviços públicos de saúde, com influência nos resultados das políticas ^13^.

Espaços com trajetórias fortalecidas pelo ambiente institucional do funcionamento asilar, associados a práticas, processos de trabalho e formativos deslocados da perspectiva interdisciplinar em rede representam entraves para incorporação de abordagens comunitárias, conforme percebido nos casos estudados ^25^. Assim, a introdução da perspectiva de direitos humanos à saúde mental é marcada por tensões, com destaque aos conflitos dada a sua complexidade do ponto de vista social ^28^, embora existam exceções ^29^.

Apesar de disparidades nas definições normativas, os resultados mostram que as experiências coincidem na dificuldade de adaptação das práticas de cuidado a uma abordagem de respeito pelos direitos humanos ^7^ ^,^ ^30^ ^,^ ^31^. Eles precisam de políticas formativas e reformas curriculares mais efetivas na direção de novos modelos de atenção psicossocial ^31^ ^,^ ^32^ ^,^ ^33^. Ainda é importante agregar aos processos formativos um olhar atento às responsabilidades técnicas e sanitárias e às singularidades dos sujeitos ^34^. Ademais, as condições laborais dos profissionais possuem descompassos com o elevado compromisso que requer o trabalho comunitário. É preciso ultrapassar a escassez de vínculos sólidos da força de trabalho, inclusive para atuar na demanda crescente de saúde mental, especialmente após a pandemia ^8^.

No Brasil, a fragilidade institucional dos serviços territoriais é realidade, visto a redução crescente do financiamento da união, em um sistema de saúde cronicamente subfinanciado ^35^, associado a um contexto de sobrecarga dos municípios com receitas próprias para saúde ^36^. Assim, as realidades locais se mostraram suscetíveis a digressões importantes, quando políticas estratégicas sofreram mudanças ^37^. Na Argentina o investimento público da União tem sido esporádico e precário em termos de quantidade e de continuidade. Assim, a escassez de recursos não só impede avanços esperados a partir da institucionalização de uma política nacional, mas também limita a sobrevivência dos serviços substitutivos já implementados ^38^ ^,^ ^39^.

Quanto às redes de atenção, as Redes de Atenção Psicossocial (RAPS) representam uma inovação na atenção ofertada aos usuários do sistema ^40^. A extensão da rede se manteve nos primeiros anos incorporando novos paradigmas de cuidado em saúde mental com uma abordagem multidisciplinar ^41^. Contudo, a experiência brasileira privilegiou a definição de serviços específicos ^25^ ^,^ ^26^. Conforme apresentado, insuficiências nos serviços e desarticulação da rede são obstáculos percebidos por atores subnacionais. Na Argentina, esse problema vê-se agravado pela ausência de diretrizes claras no nível nacional e da própria fragmentação do sistema de saúde ^42^. Portanto, apesar de se falar de uma “rede integrada de saúde mental”, e da criação de fato de algumas alternativas com enfoque comunitário, o fechamento de serviços monovalentes não acompanhou necessariamente a abertura de serviços alternativos de atenção à saúde mental, gerando o enfraquecimento do sistema. O caso de Río Negro, é uma experiência que apesar das dificuldades, é destoante em relação a outras províncias. 

Contudo, esse quadro se torna mais preocupante com a revisão das normas dos serviços de saúde mental ^43^, e da atual proposta de inclusão de serviços asilares como hospitais psiquiátricos e comunidades terapêuticas, inserida no *Decreto de Necesidad y Urgencia* ^44^, apresentado pelo atual governo de extrema-direita da Argentina. Ademais, e como resultado da fragmentação dos subsistemas públicos, privado e previdencial, os planos de saúde parecem não serem alcançados pela lei, negando serviços de saúde mental aos usuários e derivando na vulneração dos seus direitos e na sobrecarga do sistema público ^13^ ^,^ ^17^ ^,^ ^39^. No Brasil, limites para a estruturação de um sistema público universal são impostos por medidas de financeirização e fortalecimento dos planos e seguros privados, com cobertura assistencial duplicada que perpetua iniquidades de acesso ^20^.

Finalmente, observou-se, em ambos os países, uma cobertura insuficiente da demanda, decorrente de recursos (infraestrutura, econômicos e humanos) escassos e da falta de acompanhamento do crescimento populacional com a abertura de novos serviços, funcionando como um mecanismo de asfixia da rede. Pode-se somar as enormes dificuldades de diálogo e articulação entre os serviços da rede, e fora dela, e a ausência de abordagens sólidas de prevenção e no nível primário de atenção.

## Considerações finais

Há similaridades e distinções nos casos analisados, diante de condicionantes contextuais e estruturais. Diante do corporativismo segmentado do sistema de saúde argentino, limites se incidem nas trajetórias de reforma no país, com marcada heterogeneidade. No contexto brasileiro, o SUS coexiste com a expansão do setor privado, com reprodução de desigualdades. Com orientação comunitária e substitutiva ao modelo asilar, Río Negro e Ceará conformaram experiências pioneiras na área da saúde mental. A despeito das mudanças normativas, ambos os casos registram processos formativos e práticas de trabalho ainda cristalizados no modelo manicomial, com lacunas na articulação em rede e fragilidades nos mecanismos de financiamento. O estado cearense persiste com hospitais psiquiátricos especializados compondo a rede complementar à RAPS, enquanto Río Negro garantiu uma rede de saúde com serviços comunitários, com ausência de hospitais monovalentes. 

Resistências, retrocessos e avanços limitados parecem indicar que mudanças incrementais nas políticas de saúde mental alteraram, em ambos os países, as condições de implementação, distanciando seus resultados aos objetivos previamente formulados.

Como limitações do estudo, aponta-se que diferenças contextuais e estruturais entre os casos estudados limitam inferências de casualidade, predominando relações complexas e não lineares entre os fatores analisados. Vale destacar ainda possíveis vieses de seleção dos participantes da pesquisa, apesar de tentativas de diversificar os entrevistados.
